# Poster 2013: The safety of the united allergy services immunotherapy protocol

**DOI:** 10.1186/1939-4551-7-S1-P24

**Published:** 2014-02-03

**Authors:** Frederick Schaffer, Andrew Naples, Myla Ebeling, Thomas Hulsey, Larry Garner

**Affiliations:** 1 Medical University of South Carolina, United Allergy Services, Charleston, SC, USA; 2 United Allergy Services, San Antonio, TX, USA; 3 Medical University of South Carolina, Dept. Pediatrics, Charleston, SC, USA

## Background

A small, but significant number of subcutaneous immunotherapy (SCIT) based systemic reactions (SR) result in morbidity and mortality. SR range from 4 to 7% for traditional protocols and up to 34% if undergoing RUSH immunotherapy. There is estimated 1 death per 2.5 million immunotherapy injections. We report the results of an IRB approved study and contrast the safety of the United Allergy Services (UAS) SCIT treatment protocol to previously published reports. We hypothesized that a slower SCIT build up phase and pre-selection for low risk patients would prove to be safer than traditional protocols.

## Methods

A slow incremental SCIT build up phase (6 months vs. 3 months to achieve maintenance) and careful exclusion of high risk patients were salient features of the SCIT protocol utilized for 18,971 adults who were administered 1,624,135 injections and 4,643 pediatric patients (< 18 y/o) who were administered 397,466 SCIT injections for a 1 year period (2011-2012).

## Results

The adult patient SR rate per patient was 0.15% and 0.002% per injection. The pediatric patient SR rate per patient was 0.19% and per injection was 0.002%. SR rate assessment for the entire patient population was 0.16% per patient and 0.002%per injection. These results are in contrast to a reported 4% SR rate for 773 adult patients who were administered 28,000 injections (Allergy Asthma Proc. 2011;32(4):288) and up to 4.6% per injection reported for pediatric patients (Pediatrics 2013:131, 1155). The remarkably low UAS SR rates are significantly below (p < 0.0001) the cited published results. The SR for all patients are: Anaphylaxis Grade I-18, Grade II-17, Grade III-1, Grade IV-1; and for pediatric patients: Grade I-5 & Grade II- 4. Of note, no Grade V reactions (deaths) occurred in over 2 million injections.

## Conclusions

These results demonstrate the safety of the UAS immunotherapy protocol. We conclude that the UAS SCIT protocol is safe with minimal SR in comparison to previously published protocols. These safety results are due to a slower incremental SCIT build up phase and a pre-selection of low risk patients.

